# Atopic Dermatitis with Multiple Comorbidities Treated with Dupilumab. A Case Report and Review of the Literature Regarding the Safety of Dupilumab

**DOI:** 10.3390/life12101670

**Published:** 2022-10-21

**Authors:** George G. Mitroi, Loredana Elena Stoica, George F. Mitroi, Mihaela Roxana Mitroi, Cristina Violeta Tutunaru, Oana Maria Ică, Laura Simona Ianoși

**Affiliations:** 1 Department of Dermatology, Faculty of Medicine, University of Medicine and Pharmacy of Craiova, 200349 Craiova, Romania; 2 Department of Urology, Faculty of Medicine, University of Medicine and Pharmacy of Craiova, 200349 Craiova, Romania; 3 Department of Otorhinolaryngology, Faculty of Medicine, University of Medicine and Pharmacy of Craiova, 200349 Craiova, Romania

**Keywords:** atopic dermatitis, Dupilumab, safety, pregnancy, cancer, comorbidities

## Abstract

Dupilumab is the only available biological treatment for moderate-to-severe atopic dermatitis (AD). Even so, limited clinical data regarding its safety profile are available. Interactions with other drugs and the adverse effects of Dupilumab on patients with multiple comorbidities, such as chronic heart disease, diabetes, chronic kidney disease, etc., are not known yet. Moreover, there have been described cases of cutaneous lymphomas induced by Dupilumab. Therefore, the clinician that wants to start treatment for moderate-to-severe atopic dermatitis, which does not respond to conventional drugs, might be reluctant to choose biologic agents such as Dupilumab. In this paper, we reported a case of severe atopic dermatitis with multiple comorbidities in which the patient was successfully treated with Dupilumab despite numerous underlying conditions. We also conducted a review of the current literature on the safety profile of Dupilumab in special categories of patients with comorbidities, such as heart, kidney, and liver disease, oncologic conditions, and during pregnancy.

## 1. Introduction

Atopic dermatitis (AD), also called atopic eczema, affects up to 20% of children and 3% of adults [1]. AD poses a great burden to patients due to the suboptimal treatment options available at the moment. Current therapeutic agents have symptomatic effects, with no other AD-specific drugs available than Dupilumab. Pruritus represents the key feature of AD, which creates a vicious circle of pruritus—scratching—xerosis and lichenification—pruritus- scratching. The use of moisturizing ointments, topical steroids, calcineurin inhibitors, and other conventional therapies are of limited use, especially in moderate-to-severe forms of AD. The development of specific treatment options is difficult due to the complexity of the disease. AD is caused by a combination of genetic and environmental factors, which create a unique profile of each case, and this complexity of factors also provides an answer as to why there is currently no cure. AD has been associated with the activation of T-cell subsets. To summarize the pathophysiology of AD, allergens penetrate the disrupted epidermal barrier—dermal cells are triggered to produce pro-inflammatory cytokines—this produces a T cell-mediated immune response, cutaneous inflammation, and production of IL-4, IL-13, IL-31, and IL-22. In acute lesions, AD onset is characterized by profound increases in T helper cells type 2 (Th2) such as IL-4, IL-5, IL-13, IL-31 (IL = interleukin), and T helper cells type 22 (Th22), such as IL-22 and S100A protein responses [2]. Among Th2 immune mediators, IL-4 and IL-13 have been demonstrated to play a key role in AD pathogenesis. Furthermore, IL-4, IL-13, and IL-31 stimulate sensory neurons directly, resulting in pruritus [3]. Genetically, AD has been shown to be associated with IL-4 and IL-13 polymorphisms [2]. Dupilumab is the only available biologic treatment that targets IL-4 and IL-13, which are believed to be at the center of the inflammatory process in AD. Limited clinical data are available regarding the safety, interaction with other drugs, and adverse effects of Dupilumab. This paper reports a case of severe atopic dermatitis with multiple comorbidities in which the patient is successfully treated with Dupilumab despite numerous underlying conditions. We also conduct a review of the current literature on the safety profile of Dupilumab.

## 2. Case Report

**Figure 1:**
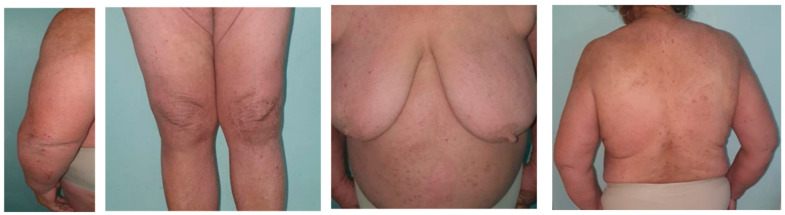
Post-grating excoriations, xerosis, hematic crusts visible on the skin.

A 52-year-old woman presented to our dermatology clinic with moderate-to-severe AD since early adulthood. The mean onset age, according to the patient, was approximately 30 years old. Previous treatments for AD included high-potency topical corticosteroids (TCS), systemic corticosteroids (SCS), and a narrowband ultraviolet-B (UVB) phototherapy, with good results upon the starting treatment but with rapid flare-ups shortly after reducing the frequency of administration/discontinuation of treatment on her own initiative. On one examination, she had diffused AD affecting approximately 68% of the body surface. Multiple papulo-nodular lesions, post-grating hematic crusts, excoriations, lichenification, and multiple post-lesional hyperpigmentations were also present on the whole-body surface (Figure 1). The dermatology life quality index score (DLQI) was 18, and SCORAD was 50.15 (she identified as having insomnia because of severe pruritus, rating both pruritus and insomnia 6 out of 10) Ultra-high-potency topical corticosteroid (clobetasol propionate) treatment was given to the patient before screening for Dupilumab initiation. The patient was known with arterial hypertension and chronic kidney disease for which she was not currently following any treatment.

Cardiological findings: congestive heart failure class III NYHA, severe degenerative aortic stenosis, hypertension gr. II, antero-superior fascicular block. Nephrology findings: chronic kidney disease stage IV, right kidney tumor. CT scan findings: right kidney: well-delimited tumor, located at the upper pole, which protrudes from the renal contour, with dimensions of 39.4/40.2 mm, an inhomogeneous structure, with areas of necrosis or hyaline matrix; the CT scan suggests renal oncocytoma without the possibility of excluding a Grawitz tumor. Laboratory findings: HbA1c 7.42% (diabetes), serum urea 84 mg/dL, serum creatinine 1.62 mg/dL, serum potassium 5.30 mmol/L. Dupilumab treatment was considered. After discussions with the nephrologist, cardiologist, and urologist, the treatment, was initiated at a loading dose of 600 mg followed by a once-every-two-week 300 mg maintenance dose along with topical emollients. No side effects occurred, and in two months, her lesions were cured completely. SCORAD improved to a value of 15.1. DLQI also improved significantly by the two months assessment, with a value of four. At a one-year follow-up, her AD remained well controlled, with 300 mg of Dupilumab being administered once every two weeks.

Nevertheless, we asked ourselves whether Dupilumab treatment was safe for such a challenging patient with multiple underlying conditions. We conducted a review of the current literature in July 2022 on the safety of Dupilumab for special categories of patients with comorbidities such as heart, kidney, and liver disease, oncologic conditions, and during pregnancy. Dupilumab is also used for the treatment of asthma, chronic rhinosinusitis with nasal polyposis, and a number of other conditions with allergic mechanisms. There have also been reports of Dupilumab being used for prurigo nodularis or bullous pemphigoid. For example, when searching for keywords such as: ”dupilumab heart” or ”dupilumab cardiovascular,” there appeared a number of articles about Dupilumab treatment for patients with heart conditions but who also suffered from asthma/prurigo nodularis. We only selected articles in which the abstract referred strictly to Dupilumab administration for atopic dermatitis.

## 3. Discussion

### 3.1. Dupilumab and Cardiovascular Conditions

A search was performed using the keywords “Dupilumab heart” and “Dupilumab cardiovascular“. Six results were chosen based on their abstracts. We found conflicting results whether AD was associated with an increased risk of cardiovascular disease. However, an association between AD-related immune biomarkers and the upregulation of cardiovascular risk-related markers has been demonstrated. Of note, one recent study showed that Dupilumab treatment might have a cardiovascular protective role by downregulating biomarkers related to atherosclerosis and cardiovascular comorbidities [3]. Villani et. al. demonstrated that Dupilumab treatment significantly modulated atherosclerosis-related genes, as did levels of other Th2-related and Th22-related markers in AD skin that were correlated with vascular inflammation [4]. Therefore, the presence of atherosclerosis should not be considered as part of the exclusion criteria. Moreover, Adachi et al. reported a case of a 43-year-old woman suffering from eosinophilic granulomatosis with polyangiitis, asthma, atopic dermatitis, and allergic rhinitis who received treatment with 300 mg of Dupilumab every 2 weeks to treat the asthma exacerbation and noted that treatment with Dupilumab was effective not only for the asthma and AD symptoms but also for the symptoms of vasculitis and heart failure [5]. Another study, which evaluated the safety of Dupilumab for 52 weeks on 62 patients aged ≥65 years suffering from AD and hypertension and other cardiovascular disorders, showed no significant adverse effects, concluding the treatment to be effective and safe for the long-term management of AD [6]. However, the study does not specify the nature of the heart conditions nor the severity. Dupilumab seems to be also safe for patients with heart transplants, as demonstrated in three case reports [7,8,9]. In the first case, a patient aged 18 with severe AD and a past medical history of left hypoplastic heart syndrome status post heart transplantation at 1 month of age, Crohn’s disease diagnosed in 2015 with no previous treatments, seasonal allergies, and asthma was initiated on Dupilumab with a loading dose of 600 mg subcutaneously followed by 300 mg every 2 weeks [7]. At the 2 years follow-up, AD remained well controlled with 300 mg of Dupilumab being administered every three weeks. The second case reported a 12-year-old girl with congenital heart disease, which required transplantation at the ages of two and seven. She received Dupilumab at a dosage of two 200 mg injections, which were supervised and continued with 200 mg every 2 weeks, with complete clearance at the 12 week follow-up [8]. The results are summarized in Table 1.

### 3.2. Dupilumab and Kidney Conditions

There are very limited data regarding Dupilumab administration to patients with kidney conditions. A search on PubMed using the keywords “Dupilumab renal“ and “Dupilumab kidney” was performed. Only seven results were found. Four articles were considered relevant for our study based on their abstract. Patruno et al. conducted a study on the long-term safety of Dupilumab on elderly patients (aged ≥ 65 years suffering from AD), which included 10 patients with kidney disease. No adverse effects were reported, and none of the patients required any Dupilumab discontinuation [6]. Of note is the fact that the study does not mention the nature or the severity of the kidney condition. Additionally, Winkler et al. reported the case of an 83 year-old patient suffering from severe chronic prurigo with chronic kidney disease stage IV, which was successfully treated with Dupilumab. Interestingly, Dupilumab treatment was chosen due to safety concerns as the first option instead of systemic steroids, methotrexate, or cyclosporine, given at the risk of nephrotoxicity and underlying renal disease [10]. This case is very interesting, as it highlights the first-hand choice of Dupilumab, rather than classical therapies, for patients with a risk of nephrotoxicity. Kha et al. reported similar results. A 32-year-old Indian man with a history of AD and an end-stage renal disease status after kidney transplantation was maintained on immunosuppressive therapy, and showed great improvement after Dupilumab administration with no adverse effects on kidney function. Dose adjustments with concurrent immunosuppressive therapies were not required [11]. On the other hand, Yamamoto et al. reported a case of a patient with AD and normal kidney function at the moment of Dupilumab initiation but who experienced a rapid decline in renal function after 9 months of treatment and was diagnosed with IgA nephropathy. The rapid decrease in renal function seemed to stop after Dupilumab discontinuation [12]. The results are summarized in Table 2.

### 3.3. Dupilumab and Liver Conditions

A search using the keywords “Dupilumab liver” on the PubMed database showed four results. Only one result was considered to be significant based on the abstract. Ludriksone et al. documented the case of a 38-year-old woman with severe AD and recurrent erythroderma under immunosuppressive therapy after a liver transplant. The patient was started on a regular dosage of Dupilumab. Neither the hepatic function panel monitorization, nor the liver ultrasound, which were performed three months after the initiation of Dupilumab treatment, showed any abnormalities. The SCORAD (Scoring Atopic Dermatitis) decreased from 81 to 25 within four weeks. No significant adverse effects were reported other than conjunctivitis developed 4 weeks after starting the treatment, which was treated successfully with glucocorticosteroid-containing eye drops [13].

### 3.4. Dupilumab and Pregnancy

We performed a search on the PubMed database using the keywords “Dupilumab pregnancy” and ”Dupilumab lactation”. Seven articles were chosen based on their abstracts.

The course of autoimmune disease during pregnancy remains a debate: some of the studies reported an improvement during pregnancy, while other reports made reference to the exacerbation of underlying conditions. Nevertheless, immune responses suffer serious alterations during pregnancy, and therefore, changes in the state of the disease are expected to occur. Patients with atopic dermatitis, asthma, and pemphigus, which are Th2- and/or Th17-dominant autoimmune diseases, usually experience the exacerbation of disease during pregnancy [14].

Dupilumab is an IgG4 antibody. Considering that IgG4 is the second most transported antibody across the placenta [15], an up-concentration in the fetus is most likely. Due to the low clinical data available, other systemic drugs for the treatment of atopic dermatitis should be used during pregnancy. However, there is neither experimental nor theoretical data to suggest Dupilumab’s teratogenic capacity [16].

The use of Dupilumab for men willing to conceive was demonstrated to be safe in a case report. Bosma et al. reported a case series of four patients (two men, two women) who wanted to conceive during Dupilumab treatment. Both men conceived successfully (with healthy partners) while receiving Dupilumab, with no fetal complications during the pregnancy. The two women involved in the study discontinued Dupilumab before conceiving [17].

Recently, four case reports have shown good maternal and fetal outcomes in pregnant patients treated with Dupilumab for atopic dermatitis. In the first case, a 36-year-old woman was started on Dupilumab with an initial dose of 600 mg, followed by 300 mg once every two weeks. Within four weeks of treatment, AD improvement was noted, but the dosage had to be decreased to 3-weekly due to eye-related adverse effects. She became pregnant 12 months later and continued to receive Dupilumab until 24 weeks and 4 days into gestation. At 37 weeks and 4 days into gestation, she gave birth to a healthy female infant via an uncomplicated spontaneous vaginal delivery [18]. A very similar case of a 33-year-old woman initiated on 300 mg of Dupilumab 300 every 2 weeks who became pregnant 12 months after treatment started was reported. The treatment was self-interrupted at 27 weeks of gestation, and a flare-up was reported 2 weeks later. Treatment was then resumed at 29 weeks into gestation and continued until 36 weeks of gestation. The patient underwent an urgent Cesarean Section at 38 weeks of gestation due to concerns for intrauterine growth restriction as well as breech position with the delivery of a healthy female infant. Upon follow-up, the infant continued to meet age-appropriate milestones. The patient did not suffer from another flare-up despite the discontinuation of treatment [19]. Mian et al. also found similar results. A 28-year-old woman was started on Dupilumab at 24 weeks of pregnancy with a loading dose of 600 mg, followed by 300 mg every other week. She had an uncomplicated delivery of a healthy infant at 37 weeks of gestation, and treatment was self-interrupted due to concerns regarding breastfeeding [20]. By contrast, the fourth report documented the case of a 35-year-old woman that continued Dupilumab treatment while breastfeeding with no adverse effects. The patient was using Dupilumab when she became pregnant and self-discontinued the treatment 2 weeks inti gestation. Of her own will, she reintroduced Dupilumab 3 months after interruption because of a severe flare-up. She continued the treatment throughout the pregnancy and gave birth to a healthy infant at 40 weeks of gestation. Dupilumab was continued while breastfeeding, and the next 4 months of follow-up showed no unwanted outcomes for the mother or the infant [21]. Most publications regarding the use of biologics during pregnancy are case reports, case series or observational studies. No randomized controlled studies have yet been performed. Nevertheless, a recently published review that summarized the effects of biologics during pregnancy found no evidence of unwanted outcomes on 313 patients [22]. The results are summarized in Table 3.

### 3.5. Dupilumab and Cancer

Clinical trials for Dupilumab did not include patients with cancer. Current immunosuppressive agents used for the treatment of atopic dermatitis raise safety concerns when being used for patients with malignities, given that there is a risk of propagating their conditions. We performed a search on the PubMed database using the keywords “Dupilumab cancer” and “Dupilumab malignities”. Seventeen articles were chosen based on their abstracts. Fowler et al. documented the cases of two patients, the first of a 22-year-old with a history of melanoma, the other of a 43-year-old with a history of the human immunodeficiency virus (HIV) on antiretroviral therapy, and anal squamous cell carcinoma (SCC). Both patients experienced great improvement with Dupilumab, with no interference to their underlying conditions. The follow-up periods were 15 months and 9 months, respectively [23]. Another recent case report had similar results, presenting the case of a 56-year-old patient with a history of non-Hodgkin’s lymphoma (NHL) with multiple relapses after initial clearance but who experienced a good clinical response to Dupilumab with no side-effects noted at the 5 month follow-up [24]. None of the analyzed articles showed any risk of cancer recurrence for patients with an oncologic history after Dupilumab treatment [23,24,25]. Interestingly, one article reported new cancer development during the treatment. Three patients were used in this study (a 46-year-old, 32-year-old, and 23-year-old). The first was diagnosed with papillary urothelial carcinoma of the bladder, and the latter two with testicular neoplasm. Dupilumab treatment was suspended for only the first patient during chemotherapy. All three patients experienced good clinical outcomes [25]. However, this was the only result we found regarding cancer development during Dupilumab treatment (other than T-cell lymphomas, see section “Dupilumab and T-cell lymphomas”), so it is safe to say that this is most likely an isolated incident, with no link to the drug. Favorable outcomes have also been reported for patients with haematologic malignancy [26,27,28]. The results are summarized in Table 4. We found no article in which Dupilumab treatment resulted in cancer recurrence.

### 3.6. Dupilumab and T-Cell Lymphomas

A search on the PubMed database using the keywords ”Dupilumab lymphoma” revealed 14 articles relevant to our study.

An association between the use of biologics and the occurrence/progression of cutaneous lymphomas has been reported [29]. Out of 8364 patients in a French study, 31 (roughly 0.37%) patients developed cutaneous T-cell lymphomas (CTCL) during treatment with biologics [30].

Mycosis fungoides (MF)—a variant of CTCL, is considered a T-helper (Th) 2-related disease, occasionally inducing eosinophilia and an increase in IgE, as seen in AD. The tumor cells in the lesional skin of the tumor-stage shift to a Th2 phenotype, which is characterized by the production of IL-4, IL-5, IL-10, and IL-13 [31]. Sézary syndrome (SS)—a variant of MF, is also considered a Th2-driven disease [32]. In SS, both malignant sezary cells and benign reactionary cells have been shown to have a Th2 predominance of cytokines such as IL4, IL5, IL10, and IL13 [32]. Considering these pathogenic mechanisms, it would be expected that Dupilumab’s effect of inhibiting the Th2 response would combat the progression of both MF and SS. Therefore, the progression or development of CTCL during Dupilumab treatment is atypical.

There have been reports of patients diagnosed with CTCL after Dupilumab administration due to the misdiagnosis of AD. CTCL, especially mycosis fungoides (MF), can be difficult to clinically differentiate from AD. In addition, some authors have described a coexistence of the two conditions in the same patient, with favorable outcomes after Dupilumab administration. The prognosis of MF differs from that of AD, so it is important to differentiate the two conditions. Although Dupilumab seemed to temporarily improve pruritus and erythema, available clinical data suggest that long-term usage can lead to the worsening or progression of CTCL. In all analyzed cases in which the initial diagnosis was that of AD—based on clinical and laboratory findings—after Dupilumab treatment, the diagnosis was changed to CTCL because of unsatisfactory clinical responses, which led to repeated investigations [33,34,35,36]. These cases could be interpreted as misdiagnosed AD, which eventually proved to be CTCL, but no signs of CTCL were seen during investigations prior to the administration of Dupilumab. Additionally, most authors describe histopathological findings that were consistent with AD before treatment started with no findings to suggest CTCL. Interestingly, it would seem that Dupilumab was a trigger factor for the development of CTCL. Furthermore, we found only three cases of patients initially diagnosed with MF who received treatment with Dupilumab [36]. All three patients experienced disease progression, and two cases resulted in patient deaths. Contrary to these results, one case reported a patient diagnosed with both MF and AD had a good clinical outcome, with improvements noticed in both conditions [37]. There have also been reports of patients diagnosed with AD that developed SS following Dupilumab administration [37,38]. No improvement in skin condition was noted in these cases, whereas another case describing Dupilumab administration for a patient diagnosed from the start with SS noted a great improvement in skin disease, pruritus, and quality of life [39]. A case of anaplastic large-cell lymphoma, which developed after complete clearance of AD with Dupilumab and that led to the patient’s death, has also been reported [40]. Given the contradictory results in many studies, it still remains a debate whether Dupilumab induces the development of CTCL, opposite to the expected outcome of improving such conditions, given the pathogenesis of cutaneous lymphomas.

All articles that we found administered Dupilumab at a normal dosage (a 600 mg loading dose followed by 300 mg every two weeks). The results are summarized in Table 5.

## 4. Conclusions

There are limited available data on the use of Dupilumab in patients with other comorbidities such as renal, cardiovascular, and oncologic conditions. In this study, we found no evidence of severe side effects in AD patients, regardless of their underlying condition, that would require Dupilumab interruption or that would contraindicate the use of this drug, except the cases mentioned under ”Dupilumab and cancer” that were misdiagnosed as AD. Our results are mostly consistent with those from other reviews regarding the safety of Dupilumab administration for patients with pre-existing comorbidities. Lukac et al. reported that 9.4% (6 out of 64 patients) discontinued Dupilumab due to severe side effects [43]. Other studies also concluded that there were no significant differences between patients with comorbidities and patients suffering only from AD as regards to the effectiveness and safety of Dupilumab [44]. Additionally, Dupilumab is also the first-hand choice for most clinicians when facing a challenging patient with underlying conditions, as shown in a recent study [45]. Although side effects while using Dupilumab are frequent (approximately 71% of patients develop 1 AE), they have a mild intensity and gravity (noninfectious conjunctivitis, eosinophilia, and injection site-reactions are the most frequently described AEs) [46]. Nevertheless, the use of Dupilumab for severe AD should not be avoided, given the portfolio of other systemic therapies used in AD, such as cyclosporine, methotrexate, and azathioprine, all with known severe side effects.

However, Dupilumab, in most patients with lymphoma misdiagnosed as AD, had in some cases severe outcomes, including death. This drug does not seem to be beneficial for CTCL, as the progression of the lymphoma was observed in most cases. Thus, whenever a case of severe AD is apparent, especially in a case of the rapid progression of the cutaneous lesions, aa diagnosis of CTCL should be taken into consideration and a subsequent pathological examination should be performed to exclude the possibility of CTCL misdiagnosis/occurrence.

As such, the outcomes, adverse or otherwise, should be reported to increase our understanding of the effects of Dupilumab use, especially for cases of AD in coexistence with other conditions.

## Figures and Tables

**Table 1:** Dupilumab and cardiovascular conditions.

Article	Age/Sex	Underlying Condition	Dupilumab Dosage	Outcome
Adachi S. et al. [5]	43/F	Bronchial asthma Allergic rhinitis Eosinophilic granulomatosis with polyangiitis	300 mg every 2 weeks	Favourable clinical outcome Improvement of AD Improvement of vasculitis symptoms
Sklover L. et al. [7]	18/M	Crohn’s disease Asthma Left hypoplastic heart syndrome status post heart transplantation at 1 month of age	Loading dose of 600 mg followed by 300 mg once every 2 weeks	Favourable clinical outcome Improvement of AD Well controlled with dupilumab 300 mg triweekly Notable improvement in quality of life
Jamgochian M. et al. [8]	12/F	Congenital heart disease requiring transplantation at age two and age seven	Two 200 mg injections and continued with 200 mg every 2 weeks	Favourable clinical outcome Complete clearance on 12-week follow-up
Hamid R.N. et al. [9]	29/F	Heart transplat at 25-years-old	300 mg once every 14 days	Favourable clinical outcome Complete clearance within 3 months

**Table 2:** Dupilumab and kidney conditions.

Article	Age/Sex	Underlying Condition	Dupilumab Dosage	Outcome
Winkler J.K. et al. [10]	83/M	Chronic kidney disease stage IV	Not specified	Favourable clinical outcome
Kha C. et al. [11]	32/M	End-stage renal disease status after kidney transplantation	Not specified	Favourable clinical outcome Improvement of AD
Yamamoto M. et al. [12]	55/F	None	300 mg every other week	Development of glomerulonephritis Rapid renal deterioration

**Table 3:** Dupilumab and pregnancy.

Article	Age	Treatment with Dupilumab before Conceiving	Dupilumab during Gestation	Continuation of Treatment during Pregnancy	Dupilumab While Breastfeeding	Maternal and Fetal Outcome
Bosma A.L. et al. [17]	29	36 weeks of treatment—discontinued—conceived 34 weeks later	No	No	No	Favourable maternal and fetal outcome.
Bosma A.L. et al. [17]	31	25 weeks of treatment—discontinued—conceived 16 weeks later	No	No	No	Favourable maternal and fetal outcome
Lobo Y. et al. [18]	36	16 months of treatment before conceiving	Yes	She continued to receive dupilumab until 24 weeks and 4 days gestation	No	Severe flare of AD after discontinuation Good fetal outcome
Akhtar N.H. et al. [19]	33	12 months of treatment before conceiving	Yes	Patient self-discontinued dupilumab at 27 weeks gestation—severe flare 2 weeks later—the patient self reinitiated dupilumab at 29 weeks gestation—remained on dupilumab until 36 weeks of gestation	No	At 38 weeks gestational age, ultrasound imaging revealed intrauterine growth restriction as well as breech position—Patient underwent Caesarean Section— Delivery of a healthy female infant
Mian M. et al. [20]	28	No	600 mg followed by 300 mg every other week (started at 24 weeks of pregnancy)	Until giving birth	No	Favourable maternal and fetal outcome
Kage P. et al. [21]	35	8 months of treatment before conceiving	Treatment was discontinued in the second week of pregnancy	Dupilumab was resumed 1 month later because of a severe flare-up	Yes	Good clinical outcome during the 4 months of observational period after giving birth

**Table 4:** Cancer patients treated with Dupilumab.

Article	Age (Years)	Cancer History	Follow Up Period (Months*/Weeks)	Adverse Effects **	Scoring System at Presentation	Scoring System after Dupilumab	Outcome	Dupilumab Dosage
Fowler E. et al. [23]	22	Melanoma	18 *	None	EASI = 62 NRS 10/10	EASI = 1.8 NRS 3/10	Improvement of AD	Not specified
Fowler E. et al. [23]	43	Human immunodeficiency virus (HIV) and anal squamous cell carcinoma (SCC)	9 *	None	NRS 10/10	NRS 4/10	Improvement of AD	Not specified
Qiu Y. et al. [24]	56	Non-Hodgkin’s Lymphoma	5 *	None	SCORAD = 57.5 EASI = 31.4 NRS 10/10	SCORAD = 12.2 EASI = 3.4 NRS 0/10	Improvement of AD	Normal dosage **
Siliquini N. et al. [25]	25	stage IIb Hodgkin’s lymphoma	54 weeks	None	Not specified	Not specified	Improvement of AD	Not specified
Siliquini N. et al. [25]	56	ductal breast carcinoma	54 weeks	None	Not specified	Not specified	Improvement of AD	Not specified
Siliquini N. et al. [25]	64	lobular carcinoma in situ and pT2pN0 infiltrating lobular carcinoma of the breast	54 weeks	None	Not specified	Not specified	Improvement of AD	Not specified
Siliquini N. et al. [25]	77	adenocarcinoma of the large intestine	54 weeks	None	Not specified	Not specified	Improvement of AD	Not specified
Siliquini N. et al. [25]	40	papillary thyroid carcinoma	54 weeks	None	Not specified	Not specified	Improvement of AD	Not specified
Siliquini N. et al. [25]	60	papillary urothelial bladder carcinoma	54 weeks	None	Not specified	Not specified	Improvement of AD	Not specified
Siliquini N. et al. [25]	53	stage IV lung adenocarcinoma	54 weeks	None	Not specified	Not specified	Improvement of AD	Not specified
Siliquini N. et al. [25]	46	papillary urothelial carcinoma of the bladder (ongoing)	Not specified	None	Not specified	Not specified	Improvement of AD	Not specified
Siliquini N. et al. [25]	32	testicular neoplasm	Not specified	None	Not specified	Not specified	Improvement of AD	Not specified
Siliquini N. et al. [25]	23	testicular neoplasm	Not specified	None	Not specified	Not specified	Improvement of AD	Not specified
Maglie R. et al. [26]	50	EDHM, lymphocytic lymphoma	5	None	Not specified	Not specified	Improvement of AD	Normal dosage **
Jin A. et al. [27]	81	CLL EDHM LC	6	None	Not specified	Not specified	Complete clearance after 2 weeks	Normal dosage **
Goyal A. et al. [28]	59	CLL EDHM	6	None	Not specified	Not specified	Complete clearance after 3 doses	Normal dosage **

NRS, Numeric Rating Scale—all NRS in the table refers to pruritus severity. * Follow up period in months. ** Adverse effects = drug related or interference with underlying condition. ** Normal dosage = loading dose of 600 mg followed by 300 mg every 2 weeks.

**Table 5:** Dupilumab and T-cell lymphomas.

Article	Age/Sex	Initial Diagnosis	Response to Dupilmab	Diagnosis Post Dupilumab	Outcome
Chiba T. et al. [33]	58/M	AD	Slight improvement of AD followed by exacerbation of cutaneous lessions	MF	Dupilumab discontinuation. MF treatment initiation
Miyashiro D. et al. [34]	51/F	AD	Slight improvement of pruritus, worsening of cutaneous lesions with tumor appearance	MF	Dupilumab discontinuation Treatment started with acitretin and PUVA with partial response
Russomanno K. et al. [35]	43/M	AD	Slight improvement in pruritus, worsening of skin eruption	AD, MF	Dupilumab discontinuation Progression of MF
Espinosa M.L. et al. [36]	64/M	AD	BSA and pruritus improvement development of erythroderma (BSA 95%), impetiginization	CTCL	Dupilumab discontinuation, improvement with radiation, bexarotene, and interferon
Espinosa M.L. et al. [36]	72/M	AD	BSA and pruritus improvement Exacerbation of cutaneous lessions	MF	Dupilumab discontinuation, treatment started with BUVB and topical corticosteroids
Espinosa M.L. et al. [36]	59/F	AD	BSA and pruritus improvement Onset of fatigue and weight loss	MF, AD	Dupilumab continued at longer intervals
Espinosa M.L. et al. [36]	40/F	AD	BSA improvement Development of erythroderma	MF	Dupilumab discontinuation, improvement with prednisone, triamcinolone, methotrexate, and NBUVB
Espinosa M.L. et al. [36]	67/M	MF	BSA and pruritus improvement	MF/SS	Dupilumab discontinuation, Disease progression, death
Espinosa M.L. et al. [36]	58/M	MF	Slight improvement of BSA worsening pruritus	MF/SS	Dupilumab discontinuation Disease progression, death
Espinosa M.L. et al. [36]	77/F	MF	No favourable response Development of erythroderma	MF/SS	Dupilumab discontinuation Disease progression
Lazaridou I. et al. [37]	37/F	AD	No response	SS	Dupilumab discontinuation, treatment started with mogamulizumab with favourable results
Lazaridou I. et al. [37]	55/M	AD/MF	Improvement in pruritus and partial remission of MF	AD/MF	Favourable outcome at 4 months of follow-up
Umemoto N. et al. [38]	48/F	AD	No response	SS	Dupilumab discontinuation Treatment started with topical corticosteroid, narrowband ultraviolet B and systemic vorinostat therapies, with good response
Steck O. et al. [39]	74/F	SS	Marked improvement of skin disease, pruritus, and quality of life	SS	Reduction in size of internal lymph nodes, increase of malignant T cells in the blood
Du-Thanh A. et al. [40]	50/F	AD	AD complete response, occurence of a new painful, erythematous and ulcerated plaque on her right breast with a diameter of 5 cm	Anaplastic large-cell lymphoma	Dupilumab discontinuation, polychemotherapy was initiated. Case resulted in patient death
Hashimoto M. et al. [41]	47/F	AD	Flat nodules developed on her cheeks	MF	Dupilumab discontinuation A combination treatment of narrow-band ultraviolet B (UVB) irradiation and oral bexarotene was started with favourable outcome
Tran J. et al. [42]	64/M	AD	Worsening off skin condition: Erythrodermic rash covering 95% of body	PSO	Dupilumab discontinuation Flow cytometry performed due to persistent erythroderma showed SS

## Data Availability

Not applicable.

## Abbreviations

AD	atopic dermatitis
BSA	body surface area
CLL	chronic lymphocytic leukemia
CT	computer tomography
CTCL	cutaneous T-cell lymphoma
DLQI	dermatology life quality index score
EASI	eczema area and severity index
EDHM	eosinophilic dermatosis of hematologic malignancy
F	female
HIV	human immunodeficiency virus
IL	interleukin
LC	leukemia cutis
M	male
MF	mycosis fungoides;
NBUVB	narrow-band ultraviolet B phototherapy;
NHL	non-Hodgkin’s lymphoma
NRS	Numeric Rating Scale
PSO	psoriasis
SCC	squamous cell carcinoma
SCORAD	Scoring Atopic Dermatitis
SCS	systemic corticosteroids
SS	Sezary syndrome;
TCS	high-potency topical corticosteroids
Th2 T	helper cell type 2
UVB	narrowband ultraviolet-B
